# Aberrant lipid profiles and lymphocyte counts in systemic sclerosis population, reassessing predictive value for concurrent cardiovascular diseases

**DOI:** 10.3389/fimmu.2025.1530909

**Published:** 2025-02-19

**Authors:** Ronghong Guo, Jinfang Gao, Yanli Yang, Ke Xu

**Affiliations:** ^1^ Third Hospital of Shanxi Medical University, Shanxi Bethune Hospital, Shanxi Academy of Medical Sciences, Tongji Shanxi Hospital, Taiyuan, China; ^2^ Department of Rheumatology, Shanxi Bethune Hospital, Shanxi Academy of Medical Sciences, Tongji Shanxi Hospital, Third Hospital of Shanxi Medical University, Taiyuan, China

**Keywords:** systemic sclerosis, cardiovascular disease, T helper cells, low-density lipoprotein cholesterol, risk factor

## Abstract

**Objective:**

To investigate alterations in blood lipid profiles and T cell subsets among systemic sclerosis (SSc) patients, and to assess their potential utility in predicting cardiovascular disease (CVD) risk.

**Methods:**

105 SSc patients and 80 age- and sex-matched healthy controls (HCs) were enrolled. Flow cytometry was employed to quantify T cell subsets. Multivariate logistic regression analysis investigated the association between blood lipid profile, T cell subsets, SSc occurrence, and CVD risk. Additionally, a prediction model was developed to assess the potential predictive value of CVD risk.

**Results:**

In the SSc patients, low-density lipoprotein cholesterol (LDL-C) (OR = 3.212, 95%CI = 1.132-9.113, *p*= 0.028), ESR (OR = 1.218, 95%CI = 1.086-1.367, *p*= 0.001), CRP (OR = 2.156, 95% CI = 1.393-3.338, *p* = 0.001), T helper (Th)cells (OR = 1.004, 95% CI = 1.001-1.008, *p* = 0.034) were positively correlated with the risk of SSc. Further studies found that absolute increases in Th cells in SSc patients were positively associated with the risk of CVD (OR=1.002, 95%CI=1.001-1.005, *p* =0.011) and were independent predictors of CVD risk in SSc. When Th cells exceeded 866.53 cells/μL, the risk of CVD in SSc patients was greatly increased (*p*<0.001).

**Conclusion:**

Altered lipid profiles and dysregulated Th cell expression in SSc patients, with a significant elevation of Th cells specifically noted in SSc-CVD patients, suggesting that Th cells may serve as a potential predictive biomarker for CVD in SSc patients, thereby aiding in early diagnosis. The underlying mechanism of this association requires further investigation.

## 1 Introduction

Systemic sclerosis (SSc) is a multisystem and chronic autoimmune disorder characterized by vasculopathy, inflammation, and tissue fibrosis (1). There are four major disease presentations: localized cutaneous SSc (lcSSc), diffuse cutaneous SSc (dcSSc), sine scleroderma, and SSc overlap syndrome (2). Skin thickening and hardening is prominent clinical manifestations, often accompanied by Raynaud’s phenomenon, arthritis, gastrointestinal involvement, interstitial lung disease, pulmonary arterial hypertension, cardiac involvement, and others (3). Epidemiological surveys indicate that the overall prevalence of SSc has reached 17.6 per 100,000 individuals, while the total incidence stands at 1.4 per 100,000 person-years; furthermore, the incidence and prevalence rates among women are fivefold higher to men (4). Additionally, epidemiological studies have revealed reduced survival rates in SSc patients, exhibiting a pooled standardized mortality rate ranging from 2.7 to 3.5. Notably, cardiovascular disease (CVD) was the primary cause of decreased life expectancy and mortality in these individuals (5). The typical representatives of CVD encompass hypertension, coronary heart disease, arrhythmia, and heart failure. Compared with healthy individuals, SSc patients exhibit a heightened prevalence of CVD, such as atherosclerosis and myocardial infarction (6). About 25% of SSc patients have experienced Raynaud’s phenomenon for more than ten years, causing delays in diagnosis and treatment. Therefore, identifying biomarkers for early detection of SSc is crucial.

The pathogenesis of SSc is intricate and not yet fully comprehended. Multiple pathophysiological mechanisms, including abnormal immune system activation, vascular injury, fibroblast proliferation, and collagen overproduction, are involved in developing SSc (7). CVD in SSc patients arises directly from autoimmune responses, vascular abnormalities, and fibrosis. It can also be triggered by interstitial lung disease and pulmonary hypertension (8). Hence, it is generally believed that SSc and CVD share a common pathogenesis. Immune cells, particularly T lymphocytes, serve as crucial serological markers in the context of SSc. The participation of T cells in the early inflammatory response through cytokine production and their interaction with fibroblasts during advanced fibrosis stages (7). The predominant T cells in this population are CD4+ T cells, commonly recognized as T helper (Th) cells. Currently, there is significant heterogeneity in the findings of studies investigating peripheral blood lymphocyte subsets in SSc patients, with several studies reporting an upward trend in Th cell populations (9). Moreover, numerous evidence suggests that the pivotal role of lipid metabolism in the pathogenesis of SSc, with alterations in lipid metabolism were considered as crucial mediators in immune cell and fibroblast activation (10). Dyslipidemia significantly promotes vascular diseases and contributes to endothelial dysfunction onset. Alterations in the lipid profile, including total cholesterol (TC), high-density lipoprotein cholesterol (HDL-C), low-density lipoprotein cholesterol (LDL-C), and triglycerides (TG), have been reported among patients with SSc compared to controls (10, 11). Close interactions between traditional cardiovascular risk factors and autoimmune response markers may contribute to the induction and progression of CVD in SSc patients. However, there is currently a lack of clinical data to evaluate whether alterations in lipid profile combined with T lymphocytes in SSc individuals are associated with the development of SSc, particularly the risk factors contributing to CVD risk associated with SSc.

In light of this, our study aims to elucidate the risk factors for SSc occurrence, and investigate the clinical predictive potential of lipid profiling for CVD risk in the specific context of immune-inflammatory responses among SSc patients. This research endeavor will facilitate clinicians’ identification of high-risk individuals and provide valuable insights for guiding treatment decisions.

## 2 Materials and methods

### 2.1 Assessments and data collection

In this retrospective study, we enrolled 105 SSc patients who fulfilled the 2013 American College of Rheumatology/European League Against Rheumatism (ACR/EULAR) classification criteria (12). These patients were initially evaluated at the Department of Rheumatology and Immunology in Shanxi Bethune Hospital from January 2018 to December 2023, and their age ranged from 18 to 80 years without receiving any specific interventions. Individuals with malignant disease, malignancy, other connective tissue diseases, and evidence of active infection were excluded. Patients using immunosuppressants, biologics, lipid-lowering drugs (statins), hypoglycemic agents, or medications associated with CVD (NSAIDs, corticosteroids) were also excluded. 80 healthy controls (HCs) without rheumatic or immune disorders were recruited from our hospital’s physical examination center during the same period, with no prior use of the medications. All subjects provided informed written consent for participation and our study adhered to the principles outlined in the Declaration of Helsinki. The Ethics Committee of Bethune Hospital, Shanxi Province (Number: NO.LYLL-2024-003/PJ03) provided approval for this study.

### 2.2 Assessments and data collection

We meticulously examined medical records and collected anthropometric and clinical characteristics of all subjects.

The basic information encompasses gender, age, body mass index (BMI), presence of hypertension, and diabetes mellitus. The duration of SSc is determined by measuring the time elapsed since the onset of the first SSc-related symptom. Skin involvement is assessed using the modified Rodman Skin Score (mRSS) (13). CVD includes coronary heart disease, essential hypertension, arrhythmias (including supraventricular, ventricular, or chronic arrhythmias), and heart failure; it is diagnosed by experienced clinicians based on established diagnostic criteria (14). Additionally, the presence of ILD was confirmed through chest high-resolution computed tomography (HRCT; GE DiscoveryRT). As invasive right cardiac catheterization serves as the gold standard for PAH diagnosis, our diagnostic criteria were based on Doppler echocardiography (15). Esophageal dysfunction was acid reflux symptoms such as heartburn or reflux esophagitis detected via gastrointestinal endoscopy. Joint involvement includes joint swelling, deformity, contracture, and tendon friction. The disease activity according to the EScSG (European Scleroderma Study Group) disease activity index (16).

### 2.3 Assessments and data collection

#### 2.3.1 Sample collection, preservation, and preparation

Concurrently with the collection of clinical data, 5.0 mL of fasting venous blood was collected from each enrolled subject upon admission. The samples were centrifuged at 3000 r/min for 5 minutes. The resulting supernatant was aliquoted into clean EP tubes. Each tube was labeled with the sample identifier, collection time, patient ID, clinical diagnosis, and intended use of the sample. Both paper and electronic records of this information were maintained.

#### 2.3.2 Preparation of reagents and instruments

The TC determination kit, TG determination kit, LDL-C determination kit, HDL-C determination kit, and CRP kit were all sourced from Beckman Coulter Experimental System Co., Ltd. For immunofluorescence, the CYTO-STAT tetraCHROME CD45-FITC/CD4-RD1/CD8-ECD/CD3-PC5 reagent with clone numbers B3821F4A, SFCI12T4D11, SFCI21Thy2D3, and UCHT1 was utilized. Item number is 6607013.

Instrumentation: A Beckman AU5800 automatic biochemical analyzer was employed. Calibration was performed using the dedicated calibrator for the Beckman Coulter biochemical analysis system, and quality control was conducted according to the established values by our hospital’s clinical laboratory department within the Beckman Coulter AU biochemical analysis system. All instruments, kits, and reagents were operated at room temperature (20-25°C). Relevant parameters of the automatic biochemical analyzer were configured accordingly. Calibration procedure: Calibrators were used with deionized water as the blank. Quality control procedures: Supporting quality control materials were used, ensuring that measured values fell within the prescribed range.

#### 2.3.3 Experimental procedures

Erythrocyte sedimentation rate (ESR) was determined using turbidimetry. C-reactive protein (CRP) levels were assessed via immunoturbidimetry, following the manufacturer’s protocol. TC were quantified using the Cholesterol Oxidase-Perioxidase Ammonium Persulfate (CHOD-PAP). TG was measured by GPO-POD (Glycerol Phosphate Oxidase- Peroxidase). And serum HDL-C and LDL-C levels were determined using the uniform phase method. Samples and reagents were added to reaction cups in precise proportions, mixed thoroughly, and incubated at 37°C for 10 minutes in a water bath. Subsequently, measurements were conducted using an automated biochemical analyzer, such as the Beckman Coulter AU5800. The TyG index was calculated according to the formula, where TyG index = Ln [triglyceride (mg/dL) * fasting blood glucose (mg/dL)/2] (17).

Two PB samples (4.5 ml each) were collected in tubes containing tri-potassium ethylenediaminetetraacetic acid (EDTA-K3) for lymphocyte immunophenotyping. The peripheral blood of patients and healthy volunteers was used to isolate PBMC and PBS, which were then adjusted to a density of 0.1-2 *10^6/100µl to prepare single-cell suspensions. Surface antibodies (CD45 for lymphocytes, CD3 for T lymphocytes, CD4 for Th lymphocytes, CD8 for Ts lymphocytes) were added followed by gentle swirling at room temperature away from light for 15 minutes. After adding 1 ml of PBS solution, the samples were centrifuged at 300g for 5 minutes. The supernatant was discarded and PBS (200-500ul) was added before testing on the machine. Flow cytometry analysis (Calibur) was performed using gated lymphocytes differentiated based on forward angular scattered light relative to lateral angular scattered light (side scatter). By utilizing a combination of two parameters, forward scatter (FSC) for size differentiation and side scatter (SSC) for particle differentiation, the removal of debris is facilitated, distinct cell populations are discerned, and lymphocyte populations are delineated. CD4 was used to distinguish CD4+ T cells from the SSC gate; 10,000 cells from the gate were taken. CD8 was used to distinguish CD8+ T cells from the SSC gate; 10,000 cells from the gate were taken. The relative percentages were obtained and analyzed using CellQuest software. The absolute number of cells in each subgroup was calculated using the following equation: absolute cell number = percentage of positive cells in each subset × the absolute number of cells (cells/μl) cells/μl whole blood. The relative percentages obtained were further analyzed using CellQuest software while BD Multitest software automatically measured the absolute numbers of T lymphocyte subsets. All immunofluorescent antibodies used in this study were purchased from BD Biosciences.

### 2.4 Statistical analysis

SPSS 26.0 (IBM, Armonk, NY, USA) software was used for statistical analysis. Normal distribution data were presented as mean ± standard deviation (SD), non-normal distribution data were presented as median and quartile [M (P25, P75)]., and counting data were expressed as percentages. When comparing the two groups of sample values, the independent sample t-test was used for normal distribution data, Mann-Whitney’s u-test was used for non-normal distribution data, and Chi-square goodness of fit test was used for counting data. Spearman rank correlation coefficient was applied for correlation analysis. Logistic regression analysis examined the association between outcome variables “SSc” and “SSC-CVD” with variables separately. A multivariate adjusted regression model was established to identify confounding factors based on a significance level of *p*<0.05. Diagnostic performance evaluation was conducted using receiver operating characteristic (ROC) curve analysis. All reported p-values were two-tailed tests and results with *p*<0.05 were considered statistically significant.

## 3 Results

### 3.1 Disposition and baseline characteristics of the patients

We comprehensively analyzed the demographic and disease-related characteristics of 105 patients diagnosed with SSc who were included in this study. The mean age was 55.64 ± 12.17 years, comprising 91 females. (The average age of the HCs was 54.38 ± 5.12 years, with a proportion of 82.5% female. There was no statistically significant difference in age and gender between the two groups.). 85 individuals (80.95%) with the diffuse type and 20 cases (19.05%) with the limited type. Anti-centromere antibody positivity accounted for 32.38%, whereas anti-Scl-70 antibody positivity was observed in 35.24%. Notably, most patients exhibited involvement of vital organs, primarily characterized by ILD which affected a substantial majority (76 cases), followed by cardiovascular diseases noted in 19 subjects. The specific results are presented in **Table 1**.

**Table 1:** Basic clinical information of the SSc population (n=105 patients).

Demographics	SSc patients (n=105)
Age, years	55.64 ± 12.17
Sex-female, n (%)	91 (86.67)
BMI, kg/m^2^	21.82 ± 3.18
Disease duration, years	7.60 ± 7.75
Diffuse cutaneous SSc, n (%)	85 (80.95)
Limited cutaneous SSc, n (%)	20 (19.05)
Modified Rodnan Skin Score, units	6 (3, 11)
Anti-centromere antibodies, n (%)	34 (32.38)
Anti-Scl-70 antibodies, n (%)	37 (35.24)
No of organs affected, n (%)	14(13.33%)
Organ involvement, n (%)	91(86.67%)
Cardiovascular diseases, n (%)	19(18.10)
Interstitial lung disease, n (%)	76 (72.38)
Pulmonary arterial hypertension, n (%)	26 (24.76)
Gastrointestinal involvement, n (%)	41 (39.05)
Arthritis, n (%)	36 (34.29)
Hypertension, n (%)	20 (19.05)
Diabetes, n (%)	6 (5.71)
Active patients with disease activity index >3 EScSG, n (%)	56(53.33)

Data represented mean ± SD or median M (P25, P75) when data were not normally distributed. BMI: body mass index; EScSG, European Scleroderma Study Group; SSc, systemic sclerosis.

### 3.2 Dyslipidemia and abnormal expression of T lymphocytes in SSc patients

**Figure 1:**
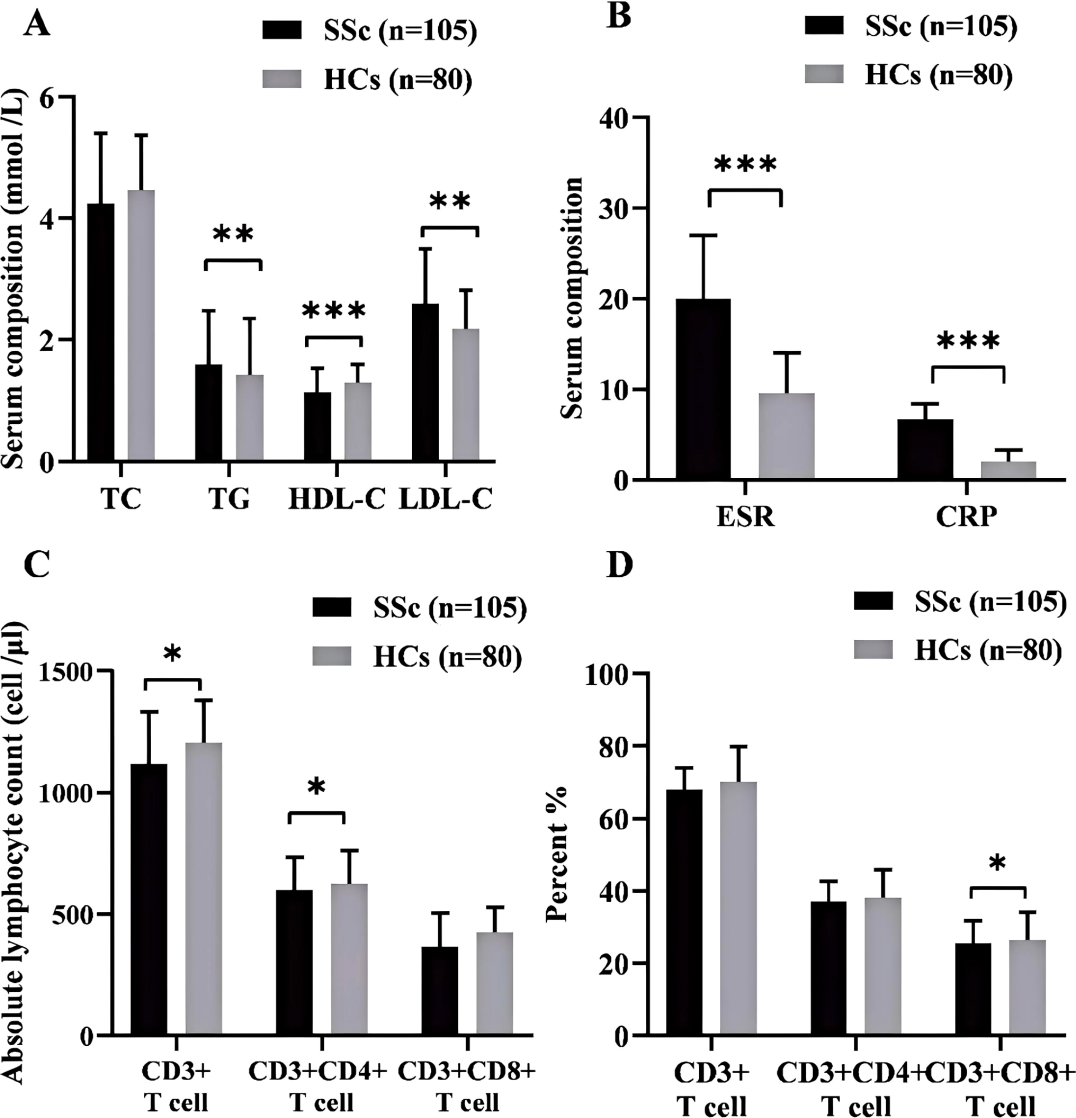
**(A)** Comparison of blood lipid profiles between SSe patients and HCs. **(B)** Inflammatory markers in SSc patients and HCs. **(C)** The absolute counts of lymphocytes, T (CD3+), Th (CD3+CD4+), and Ts (CD3+CD8+) in SSc patients and HCs. **(D)** The percentage of lymphocytes T (CD3+), Th (CD3+CD4+), and Ts (CD3+CD8+) between SSc patients and HCs. SSc, systemic sclerosis; HCs. Healthy controls; TC, total cholesterol; TG, Triglyceride; HDL-C, high-density lipoprotein cholesterol; LDL-C, low-density lipoprotein cholesterol: *p<0.05; **p<0.01; ***p<0.001.

After conducting a univariate analysis, we observed metabolic abnormalities in SSc patients compared to HCs (**Figure 1A**). The key findings included elevated levels of TG (1.60 ± 0.88 vs 1.43 ± 0.91 mmol/L, *p* = 0.002) and LDL-C (2.59 ± 0.90 vs 2.17 ± 0.64 mmol/L, *p* = 0.001) in SSc patients. Notably, the TyG index showed a significant increase (8.65[8.42, 8.90] vs 8.35 [8.08, 8.77], *p*=0.005). HDL-C levels were decreased (1.14 ± 0.88 vs 1.30 ± 0.29 mmol/L, *p <*0.001), while TC levels did not differ significantly (4.25 ± 1.15 vs 4.45 ± 0.91 mmol/L, *p*=0.073). In terms of inflammatory markers, ESR and CRP were significantly higher in the SSc group (20 [12, 36] vs 9.5[4.5, 14]mm/h, *p*<0.00l; 6.64[2.87, 22.53] vs 2 [1.14, 4.51] mg/L, *p*<0.00l) (**Figure 1B**). Subsequently, we conducted a comparative analysis of T lymphocyte levels (absolute and percentage as depicted in **Figures 1C, D**. The absolute count of T(CD3+) lymphocytes was significantly lower in SSc patients than HCs patients (982.06 [657.28, 1474.57] vs 1204 [1008.40, 1367.19] cell/μL, *p* =0.018). The absolute count of Th (CD3+CD4+) cells exhibited a significant decrease (597.36 [302.75, 824.07 vs 625.42[537.25, 760.355] cell/μL, *p* =0.034). However, the percentage of Th cells was significantly higher than that observed in HCs (41.6 [35.10, 46.89] vs 38 [32.50, 43.20] %, *p* =0.021), while there were no significant changes noted for both the absolute value and percentage of Ts (CD3+CDs+) cells.

**Figure 2:**
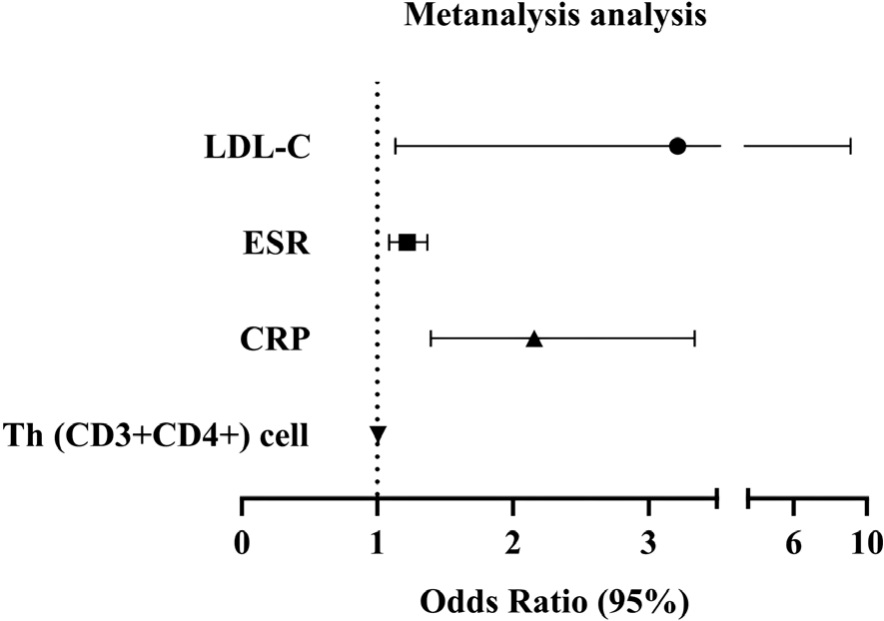
A logistics forest diagram is constructed based on Model 3. Dots and lines represent the HR and 95% CI.

The variables with a significance level of *p* < 0.05 in **Figure 1** were subjected to Multivariate Logistic regression analysis (**Table 2**, **Figure 2**) for difference comparison. In Model 1, the blood lipid profile was adjusted, revealing a negative correlation between HDL-C levels and the occurrence of SSc (OR = 0.246, 95%CI = 0.080-0.751, *p* = 0.014). Conversely, LDL-C levels showed a positive correlation with the occurrence of SSc (OR= 2.787, 95%CI =1.618-4.799, *p <*0.001). Model 2 further incorporated adjustments for ESR and CRP, demonstrating that higher ESR and CRP values increased the likelihood of SSc (OR = 1.218, 95%CI=1.086-1.367, *p <*0.001; OR=2.267, 95%CI=1.483 -3.463, *p <*0.001), except for the effect on LDL-C. After the final correction in model 3 revealed that risk factors for SSc included LDL-C (OR = 3.212, 95% CI = 1.132-9.113, *p* =0.028), ESR (OR = 1.218, 95% CI = 1.086-1.367, *p* = 0.001), CRP (OR = 2.156, 95% CI = 1.393-3.338, *p* = 0.001), Th (CD3+CD4+) cell (OR = 1.004, 95% CI = 1.001-1.008, *p* = 0.034). The identical outcome is illustrated in **Figure 2**.

**Table 2:** SSc risk-related multimodel regression analysis with multivariate adjustment.

Variables	Beta	OR (95% CI)	*p* Values
Model 1^a^			
Constant	-2.643		
HDL-C, mmol/L	-1.404	0.246 (0.080-0.751)	**0.014**
LDL-C, mmol/L	1.025	2.787 (1.618-4.799)	**<0.001**
Model 2^b^			
Constant	-9.691		
LDL-C, mmol/L	1.280	3.596 (1.358-9.526)	**0.010**
ESR, mm/h	0.179	1.196 (1.084-1.320)	**<0.001**
CRP, mg/L	0.818	2.267 (1.483-3.463)	**<0.001**
Model 3^c^			
Constant	-11.154		
LDL-C, mmol/L	1.167	3.212 (1.132-9.113)	**0.028**
ESR, mm/h	0.197	1.218 (1.086-1.367)	**0.001**
CRP, mg/L	0.768	2.156 (1.393-3.338)	**0.001**
Th (CD3+CD4+) cell, cell/μL	0.002	1.004 (1.001-1.008)	**0.034**

The Bolded value represents statistical significance. Model1^a^ was adjusted according to cholesterol, HDL-C, and LDL-C. Model 2^b^ was further adjusted for ESR and CRP. Model 3^c^ was based on Model 2. The continuous variables in **Figure 1** include the absolute value of T (CD3+) cells and Th (CD3+CD4+) cells, and the percentage of Th (CD3+CD4+) cells for further adjustment. OR: Odds Ratio; CI: Confidence Interval; ESR, Erythrocyte sedimentation rate; CRP, C-reactive protein; HDL-C, high-density lipoprotein cholesterol; LDL-C, low-density lipoprotein cholesterol Bold values mean statistical significance.

### 3.3 Lipid levels and T-lymphocyte subsets exhibit an association with the risk of CVD in Systemic sclerosis patients

The study enrolled 105 patients diagnosed with SSc and categorized them into two groups based on cardiovascular disease (see **Table 3**). No significant differences were observed between SSc patients with and without CVD regarding sex, age, BMI, and presence of diabetes. However, the incidence of combined hypertension was higher in SSc-CVD patients compared to those in SSc-non-CVD (47.37 vs 88.37%, *p* =0.002). Univariate analysis revealed significantly elevated TG levels in SSc-CVD patients compared to the SSc-non-CVD group (2.23 ± 1.62 vs 1.46 ± 0.53 mmol/L, *p* =0.002). Moreover, the TyG index also exhibited a significant increase (8.94 [8.80,9.13] vs 8.57 [8.36, 8.82], *p* =0.001), while no significant differences were found for HDL-C and LDL-C levels between the two groups. In terms of T lymphocyte lineage analysis, we specifically observed a significant increase in the absolute value of Th (CD3+CD4+) cells within the SSc-CVD group (807.46 [383.40, 1194.67] vs571.55 [285.02, 759.85] cells/μL, *p* =0.021). To further investigate independent factors associated with CVD risk among SSc patients, multiple logistic regression analysis was performed considering the aforementioned indicators (**Table 4**). The results demonstrated that Th (CD3+CD4+) cells independently influenced CVD risk, and there existed a positive correlation (OR=1.002, 95%CI=1.001-1.005, *p* =0.011).

**Table 3:** Characteristic data of SSc patients with and without CVD.

Characteristic	SSc-CVD(n=19)	SSc-non-CVD(n=86)	*p* Value
Age, years	60.24 ± 6.05	57.24 ± 12.74	0.183
Sex-female, n (%)	15 (78.95)	76(88.37%)	0.276
BMI, kg/m^2^	23.03 ± 3.69	21.58 ± 3.05	0.133
Hypertension, n (%)	9 (47.37)	11 (12.79)	**0.002**
Diabetes, n (%)	3 (15.78)	10 (11.62)	0.618
Anti-centromere antibodies, n (%)	5 (26.32)	21 (24.42)	0.862
Anti-Scl-70 antibodies, n (%)	6 (31.57)	30(35.00)	0.784
TC, mmol/L	4.27 ± 1.22	4.24 ± 1.14	0.980
TG, mmol/L	2.23 ± 1.62	1.46 ± 0.53	**0.002**
HDL-C, mmol/L	1.05 ± 0.20	1.17 ± 0.43	0.392
LDH-C, mmol/L	2.60 ± 0.76	2.59 ± 0.94	0.747
Glucose, mmol/L	5.72 ± 1.72	4.95 ± 1.17	0.053
TyG	8.94 [8.80,9.13]	8.57 [8.36, 8.82]	**0.001**
ESR, mm/h	26.00[12.50,47.50]	20.00 [12.00, 33.00]	0.198
T (CD3+) cell, cell/μL	1156.20 [810.13, 1862.84]	964.50 [637.29, 1373.40]	0.245
Th (CD3+CD4+) cell, cell/μL	807.46 [383.40,1194.67]	571.55 [285.02, 759.85]	**0.021**
Ts (CD3+CD8+) cell, cell/μL	427.50 [300.37, 514.45]	323.07 [242.52,558.25]	0.690
T (CD3+) cell, %	74.18[68.62, 77.98]	73.24[67.40, 78.33]	0.987
Th (CD3+CD4+) cell, %	44.00[39.53, 50.76]	40.75[35.02, 46.01]	0.198
Ts (CD3+CD8+) cell, %	23.27[14.23, 31.68]	30.91[21.88, 34.88]	0.079
mRSS score	8.00[4.50, 10.00]	6.00[3.00,12.00]	0.604

Data represent mean ± SD or median M (P25, P75) when data were not normally distributed. BMI, body mass index; TC, total cholesterol; HDL-C, high-density lipoprotein cholesterol; LDL-C, low-density lipoprotein cholesterol; TG, Triglyceride; ESR, Erythrocyte sedimentation rate; mRSS score, modified Relative Severity Score; Bold values mean statistical significance.

**Table 4:** Risk factors of SSc-CVD upon logistic multivariate regression.

Variables	Beta	OR (95% CI)	*p* Values
TG	0.268	1.307 (0.147-11.654)	0.810
TyG	1.467	4.335 (0.627-29.375)	0.461
Th (CD3+CD4+) cell	0.002	1.002 (1.001-1.005)	**0.011**
Hypertension 0			
1	-1.149	0.317 (0.047-2.130)	0.238

OR, Odds Ratio; CI, Confidence Interval; TyG, triglyceride-glucose index; TG, Triglyceride; CVD, cardiovascular disease; Bold values mean statistical significance.

### 3.4 Potential predictive value of T helper cells for CVD risk in SSc patients

**Figure 3:**
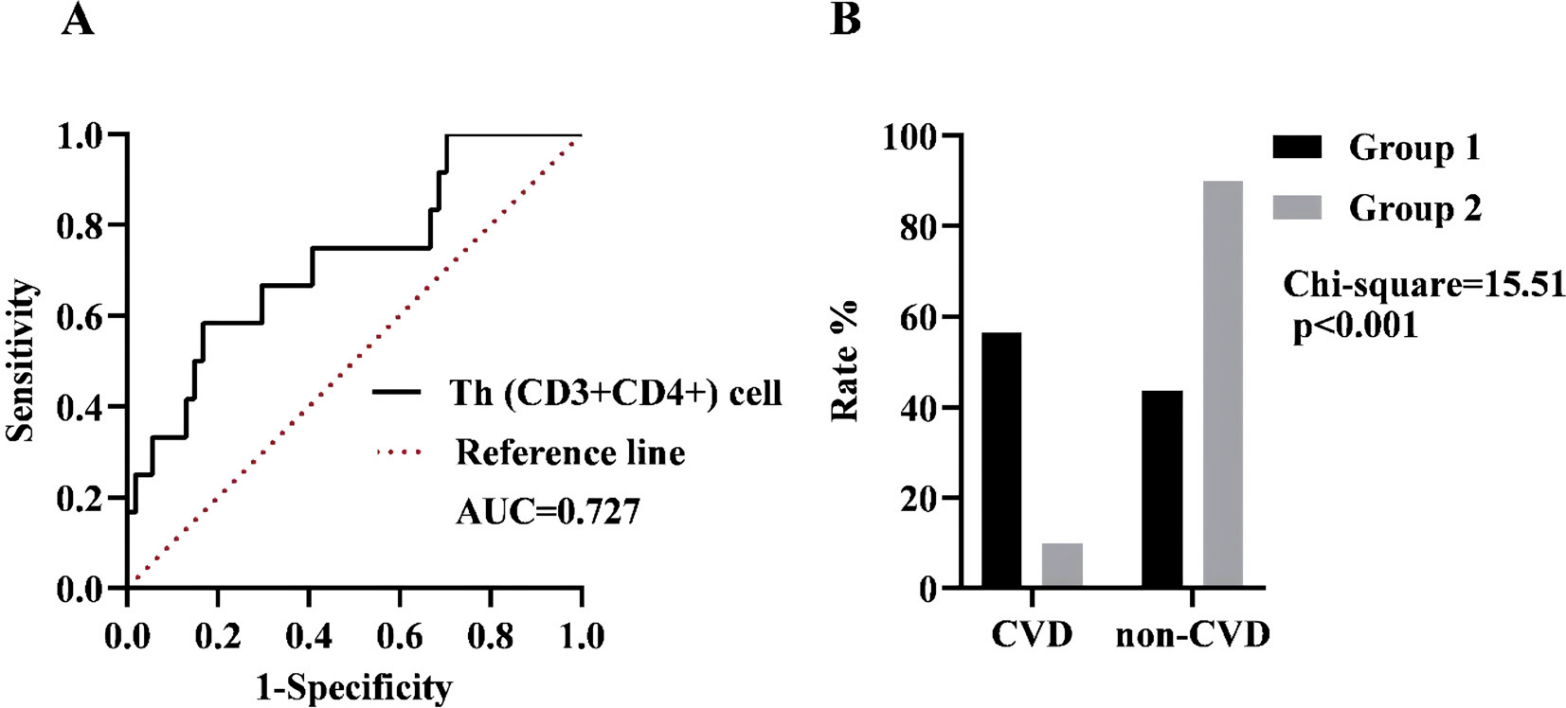
**(A)** ROC curve analysis in the presence of CVD. Black line: Th (CD3+CD4+) cell, AUC = 0.727, 95% CI = 0.565-0.890, *p*=0.015. Dotted line: Reference line. **(B)** The incidence of the two groups was compared based on the optimal cut-off value. Group 1 was defined as having a cell count of more than 866.53 cells/µL, while Group 2 was defined as having a cell count of less than 866.53 cells/µL.

We further investigated the predictive capacity of Th (CD3+CD4+) cells in assessing the risk of CVD in SSc patients, based on the presence of “CVD” status. As depicted in **Figure 3A**, we identified an optimal cut-off point for Th (CD3+CD4+) cells at 866.53 cells/μL (sensitivity of 58.33% and specificity of 83.33%). The Yoden index was calculated to be 0.417, indicating the high accuracy of our model in effectively predicting CVD risk among SSc patients. By validating the diagnostic accuracy of this threshold (**Figure 3B**), we observed SSc patients had a substantial increase in CVD risk when the Th (CD3+CD4+) cell count exceeded 866.53 cells/μL (*p <*0.001).

## 4 Discussion

In this study, we detected abnormal blood lipid levels and T lymphocytes in SSc patients, multivariate analysis revealed a significant association between serum LDL-C levels and both Th cell activity and the presence of SSc. Furthermore, we conducted a comprehensive analysis of the CVD risk factors in SSc patients and concluded that immune factors, specifically Th cells, hold potential predictive value for its occurrence.

Certain autoimmune diseases (ADs), such as rheumatoid arthritis (RA) (18), systemic lupus erythematosus (SLE) (19), and psoriasis (20), were associated with elevated morbidity and mortality from CVD. These conditions contribute to a chronic systemic inflammatory state characterized by pro-inflammatory cytokines and autoantibodies, which play a crucial role in the pathogenesis of CVD. A large population-based study confirmed that individuals with ADs have a higher CVD risk, and cardiovascular risk progressively increases with the number of coexisting ADs. Notably, SSc was identified as having the highest overall cardiovascular risk among common ADs included in the study (21). SSc patients exhibited an elevated risk of CVD and a higher prevalence of other cardiac manifestations, including pacemaker implantation (22). In our study, the incidence of CVD in SSc patients was found to be 18.10%. We postulated that this increased risk may be attributed to two main factors: firstly, the underlying pathology and pathogenesis of SSc involving repetitive focal ischemia caused by microangiopathy triggers aberrant immune responses, ultimately leading to myocardial fibrosis (23); secondly, it is widely believed that the heightened risk of CVD can be attributed to well-established common risk factors, including but not limited to age, race, systolic blood pressure, cholesterol level, diabetes, dietary habits, obesity, sedentary lifestyle (24). This study highlights the interaction between chronic inflammation and traditional CVD risk factors as its core focus.

Previous studies have demonstrated conflicting findings regarding the lipid profile in SSc patients. Firstly, our findings demonstrated that SSc patients exhibited significantly lower levels of HDL-C. Kwon et al. reported a negative association between the incidence of SSc and higher levels of TC, HDL-C, and LDL-C, with a 6.4% decrease in SSc incidence for every 10 mg/dL increase in HDL-C level (10). Another study revealed that dcSSc and lcSSc patients exhibited lower serum HDL-C levels, elevated TC levels, and increased atherosclerosis index compared to controls (25). Interestingly, a significant association was observed between reduced levels of HDL-C and the presence of anti-centromere antibodies as well as pulmonary hypertension. At the same time, no correlation was found with ESR or CRP levels (26). Furthermore, our study revealed that LDL-C levels were significantly elevated and positively correlated with SSc. The metabolomic analysis of plasma in SSc patients revealed a significant down-regulation of lysophosphatidylcholine (LPC), which is a major constituent of oxidized low-density lipoprotein (oxLDL). It exerts effects on oxidative stress, endothelial cells, and lymphocytes, thereby eliciting the activation of pro-inflammatory cytokines (27). Conversely, LDL levels were elevated in SSc patients with ILD in a cohort study. A similar phenomenon was observed in model mice, demonstrating that LDL induces apoptosis and TGF-β1 production (28). TG serves as the primary source of human energy, although there is no consensus on the TG levels among SSc patients. However, SSc patients exhibited significantly elevated TG levels compared to the control group (29). Gogulska, Z et al. also reported similar findings, demonstrating elevated TG levels in SSc patients (11). This observation may be attributed to reduced lipoprotein lipase (LPL) activity and the influence of anti-LPL antibody formation (30). In this study, apart from analyzing lipid spectrum abnormalities in SSc patients, we also incorporated inflammatory indicators into the multi-factor analysis. CD4+ and CD8+ T cells, the predominant T cell subsets detected in both serum and skin biopsies during the inflammatory response and fibrosis stage of SSc patients, play a crucial role in activating angiogenesis, promoting collagen production, and inducing fibrosis. Specifically, Th (CD4+) cells and their associated cytokine dysregulation exhibit distinct roles across various stages of diseases (31). After adjusting for multiple confounding factors, our analysis concluded that ESR, CRP, and Th cells are associated with the occurrence and development of SSc. This suggested that T lymphocyte subsets do not act independently but rather contribute to immune responses through interactions within related immune regulatory networks or pathways. Furthermore, our data indicated that LDL-C levels along with Th cells could serve as non-causal biomarkers associated with SSc events and may be useful for identifying individuals at a heightened risk for developing SSc.

SSc patients exhibited unfavorable lipoprotein profiles and an increased CVD risk (11). Therefore, this study further investigated the alterations in lipid levels and T cell populations in SSc patients with concomitant CVD. Through univariate analysis, we observed that SSc-CVD patients exhibited significantly elevated TG levels compared to SSc patients without CVD. Ferraz-Amaro, I. et al. previously reported dyslipidemia and reduced cholesterol effector capacity (CEC) in SSc patients, considering the association between decreased CEC and CVD risk, they suggested that reduced CEC might contribute to augmented atherosclerotic burden in SSc patients (32). Previous investigations have also found no increase in the incidence of traditional CVD risk factors such as obesity, hyperlipidemia, hypertension, and diabetes among SSc individuals (33, 34), which aligns with the findings obtained from our study. Compared with traditional cardiovascular risk factors, Th cells were found to be elevated in SSc-CVD patients in our study. Similarly, a cohort study observed an excess risk of CVD associated with ADs that could not be attributed to conventional risk factors such as age, gender, and socioeconomic status. These findings suggested that inhibiting chronic inflammation may reduce the incidence of cardiovascular events independently of lipid modification or other risk factors (21). Studies have also demonstrated that high concentrations of proinflammatory molecules, including tumor necrosis factor-alpha (TNF-α) and interleukin-6 (IL-6), contribute to the accelerated development of atherosclerosis and cardiovascular events in patients with RA and SLE (35). However, limited research has been conducted on SSc. We proposed that Th cells, as inflammatory mediators, play a significant role in the occurrence and progression of SSc complicated with CVD.

Subsequently, we analyzed to elucidate the potential pathogenesis underlying alterations in lipid metabolism and T lymphocytes observed in SSc-CVD individuals. Firstly, a hallmark feature of SSc is the activation of immune cells and the formation of autoantibodies. Chronic systemic inflammation plays a pivotal role in the development and progression of SSc-CVD (36). Faccini et al. reported that SSc patients exhibit underlying coronary microvascular dysfunction, which may precede coronary atherosclerosis by several years and is associated with systemic inflammatory responses (35). Additionally, even relatively modest elevations in inflammatory markers such as CRP have been shown to predict cardiovascular events in the general population (37). B cells, T cells (including clonally expanded CD4+ cells with heightened proinflammatory and CD8+ cells with cytotoxic properties), granzyme B, TNF-α, IL-6, and TLR 2 and 4 all contributed to the inflammatory processes underlying atherosclerosis (38). Given the extensive research and discussion on the functional role of T cells in CVD, it has been observed particularly that Th17 cells and other pro-inflammatory T cell subsets promoted atherosclerosis, Tregs possessed inhibitory effects. Notably, a common immunomodulatory mechanism has been identified in the investigation of SSc-associated CVD (39). Secondly, SSc is distinguished by characteristic vascular alterations. Endothelial dysfunction was central to the pathophysiology of SSc and atherosclerosis and was closely linked to oxidative stress-induced production of reactive oxygen species (ROS). ROS oxidized LDL, led to the formation of ox-LDL, which was consumed by monocytes and ultimately resulted in the development of atherosclerotic foam cells (40). Emerging studies have identified autoantibodies that may contribute to atherogenesis, including elevated levels of circulating ox-LDL/β2-glycoprotein 1 complexes and anti-ox-LDL antibodies in SSc patients (34). Additionally, the anti-centromere and cardiolipin antibodies have been observed in atherogenesis (37, 41). The interplay between inflammation and lipids appears to play a crucial role in the pathogenesis of SSc-CVD. HDL-C effectively eliminated cholesterol in peripheral cell membranes, leading to decreased expression of Class II major histocompatibility complex molecules and ultimately reducing T cell activation. Consequently, diminished HDL-C levels might elevate the risk of SSc development through impaired immune regulation, elucidating the negative correlation between HDL-C levels and SSc incidence (42). Numerous studies have demonstrated that cholesterol can induce apoptosis, inflammation, epithelial-mesenchymal transition (EMT), and fibrosis. Hypercholesterolemia promoted cholesterol accumulation in macrophages and other immune cells, resulting in an inflammatory response characterized by increased toll-like receptor (TLR) signaling, inflammasome activation, as well as enhanced monocyte and neutrophil production in bone marrow and spleen. At the cellular level, TLR signaling activation reduced CEC, leading to further intracellular cholesterol accumulation and the inflammatory response amplifying. Therapeutic interventions targeting this pathway may disrupt the link between cholesterol accumulation and inflammation, potentially benefiting metabolic disorder individuals (43).

Given the potential significance of CVD in the long-term prognosis of SSc patients, accurate prediction is crucial. However, consensus guidelines on SSc-CVD screening, imaging techniques utilization, and appropriate management of various cardiac injuries in SSc (ranging from subclinical to overt manifestations involving myocardium, conduction system, and pericardium) heavily rely on expert opinions (44). Common noninvasive tests such as Doppler echocardiography and MRI are utilized for the assessment of cardiac anatomy and function (45). Therefore, it is imperative to identify biomarkers that can simplify diagnosis processes while evaluating treatment outcomes and predicting recurrence rates. To achieve this, we conduct clinical studies to comprehensively investigate risk factors and predictors of heart injury to stratify and monitor patients. Based on our study design and the aforementioned results, Th cells have been identified as reliable predictors of SSc-CVD, with an optimal threshold value of 866.53 cells/μL determined through ROC curve analysis. Consequently, detecting Th cell levels exceeding this threshold in SSc patients during clinical assessments should raise awareness regarding the risk of developing CVD.

The optimal management of SSc remains a significant challenge. Currently, there are no specific pharmacological interventions recommended for halting disease progression. However, the Trial and Research Group of the EULAR has published the latest consensus recommendations for the treatment of SSc, emphasizing selective medication based on organ involvement (46). Furthermore, lipid-lowering agents, such as statins and fenofibrate, have exhibited anti-inflammatory effects alongside reducing lipid levels. Multiple clinical trials have shown the efficacy of statins in treating vascular lesions by reducing endothelin-1 levels and lowering the risk of finger ulcers (47). Notably, statins have been utilized in SSc patients and have demonstrated modulation of Th1, Th2, and Th17-related cytokines production influences. Particularly simvastatin exhibited immunosuppressive effects by dose-dependently reducing the secretion levels of SSc-associated cytokines (48). The clinical application of statins in SSc patients requires further extensive clinical research, highlighting the potential role of lipid profile in SSc pathogenesis. Moreover, there is a lack of studies on immune cell therapy as a treatment option for SSc-CVD. However, this study identified Th cells as independent risk factors for SSc-CVD, thus providing valuable insights into the potential application of immune cell therapy for severe cases of SSc-CVD.

However, this study has certain limitations. Firstly, because of the low prevalence of SSc, our sample size was relatively small, particularly among SSc-CVD patients. Second, due to the retrospective nature of the study design and the omission of certain traditional CVD risk factors such as lifestyle, future studies should incorporate large-scale, multicenter data to validate these findings. In addition, this study primarily focused on assessing changes in LDL-C levels but did not evaluate ox-LDL. This omission may have led to an underestimation of LDL’s true impact on SSc-CVD. Future research should address this limitation by incorporating ox-LDL assessments to provide a more complete understanding of the role of LDL in SSc-CVD. Additionally, it would be intriguing to include patients with CVD but without SSc in our analysis. Despite these constraints, our findings remain robust as they were derived from a substantial cohort and represent the pioneering attempt at assessing the association between lipid profiles and T lymphocytes concerning SSc development.

Our study presented novel findings regarding the lipid profile and T lymphocytes and the risk of SSc and SSc-CVD. Specifically, we observed elevated levels of LDL-C and Th cells in SSc patients. Furthermore, Th cells emerged as an independent factor for predicting CVD risk in SSc patients, with a threshold value of 866.53 cells/μL indicating increased risk. We propose that Th cells have the potential as biomarkers and therapeutic targets for identifying CVD in SSc individuals.

## Data Availability

The raw data supporting the conclusions of this article will be made available by the authors, without undue reservation.
